# Effects of exposure to immersive videos and photo slideshows of forest and urban environments

**DOI:** 10.1038/s41598-021-83277-y

**Published:** 2021-02-17

**Authors:** Fariba Mostajeran, Jessica Krzikawski, Frank Steinicke, Simone Kühn

**Affiliations:** 1 Universität Hamburg, Department of Informatics, Human-Computer Interaction Group, 22527 Hamburg, Germany; 2 University Medical Center Hamburg-Eppendorf, Clinic and Polyclinic for Psychiatry and Psychotherapy, Neural plasticity Group, 20246 Hamburg, Germany; 3 Max Planck Institute for Human Development, Lise Meitner Group for Environmental Neuroscience, 14159 Berlin, Germany

**Keywords:** Psychology, Computer science

## Abstract

A large number of studies have demonstrated the benefits of natural environments on people’s health and well-being. For people who have limited access to nature (e.g., elderly in nursing homes, hospital patients, or jail inmates), virtual representations may provide an alternative to benefit from the illusion of a natural environment. For this purpose and in most previous studies, conventional photos of nature have been used. Immersive virtual reality (VR) environments, however, can induce a higher sense of presence compared to conventional photos. Whether this higher sense of presence leads to increased positive impacts of virtual nature exposure is the main research question of this study. Therefore, we compared exposure to a forest and an urban virtual environment in terms of their respective impact on mood, stress, physiological reactions, and cognition. The environments were presented via a head-mounted display as (1) conventional photo slideshows or (2) 360$^{\circ }$ videos. The results show that the forest environment had a positive effect on cognition and the urban environment disturbed mood regardless of the mode of presentation. In addition, photos of either urban or forest environment were both more effective in reducing physiological arousal compared to immersive 360$^{\circ }$ videos.

## Introduction

Nowadays, individuals spend more and more time in artificially designed living spaces, in particular, humans spend up to 90% indoors^1^. This tendency has led to an isolation of individuals from regular contact with nature which has a negative impact on their mental and physical health. Several studies have demonstrated that such artificial stimulation and being in purely human-generated environments can lead to mental fatigue as well as a loss of vitality and health^2,3^.

These negative effects can be reduced by means engaging in interactions with nature^4^. There is evidence to suggest that natural environments have a positive influence on human psychology, physiology, and cognition^5–7^. According to the Attention Restoration Theory (ART), natural environments capture less cognitive resources, and therefore, allow an interruption of attention-grabbing tasks inherent in urban environments and thus, elicit attention restoration and recovery from mental fatigue^8–10^. Natural elements such as green landscapes and flowing waters have a calming effect on physiological arousal^11,12^. One of the long-term effects of access to nature is a positive attitude towards life and an increased satisfaction with one’s own home, one’s own work and generally one’s own life^8,13^.

As an instance of natural environments, forests have been studied frequently suggesting their positive effects on human body and mind^14–19^. These positive effects include, but are not limited to psychological relief, lower stress and depression levels^19–24^ as well as physiological effects such as lower blood pressure, heart rate (HR), and salivary cortisol hormone levels^18,25,26^. Therefore, forest therapy, also referred to as “forest bathing”, is practiced widely, in particular in Asia, to derive substantial benefits from the positive health effects of walking, resting, and interacting with forests^27–34^.

For people with limited access to nature (e.g., elderly in nursing homes, hospital patients, or jail inmates), already the visual representation of nature can relieve stress and improve emotional well-being^22,35–39^. Many studies in environmental psychology have used conventional photos to compare natural and urban environments or to demonstrate the positive effects of nature photos^5,6,40,41^.

In this context, immersive virtual reality (VR) may facilitate some of these characteristics such as the feeling of being in nature during the exposure^42^. By reproducing realistic stimuli and eliciting psychological processes, VR has the potential to increase external validity of the research findings^43^. It can, in addition, provide the experimenter (and potentially therapists) with a systematic control over the natural elements such as weather conditions, vegetation (up to the smallest details such as movements of the grass and leaves on the trees), wildlife, and lighting that is hard or impossible to achieve in real life^44,45^. Furthermore, therapeutic applications may benefit from the low-cost virtual environments, which can be duplicated and distributed easily, making them usable at a larger scale^46^ and make it accessible to individuals in need, e.g., in nursing homes. Thus, VR can complement the research on human perception and behavioral responses to nature stimuli by maximizing the benefits of lab-based (e.g., control over independent variable) and field-based (e.g., realistic stimuli) experiments^43^.

For this reason, previous studies have already employed nature exposure in VR. Several studies have compared real physical nature exposure with exposure to 360$^{\circ }$ videos of nature^47–52^. For instance, Browning et al.^47^ compared real nature exposure and a 360$^{\circ }$ VR nature video recorded from the same location. In comparison to a physical indoor environment without nature, both real and VR nature exposure were more restorative and increased physiological arousal. Although, only the real exposure to nature outdoors increased mood in a positive direction.

Researchers have also compared exposure to different environments merely in VR. For instance, a study^53^ demonstrated that different types of forest environments, presented via 360$^{\circ }$ videos, can improve mood and relieve stress. Another study^54^ revealed that in comparison to a control environment, exposure to 360$^{\circ }$ videos of nature can reduce physiological arousal and negative affect. Furthermore, in a study by Chung et al.^55^ and in comparison to 360$^{\circ }$ videos of fireworks, exposure to 360$^{\circ }$ videos of nature improved cognitive functioning and restored involuntary attention of the participants^55^. In comparison to urban environments, Yu et al.^56^ could show that exposure to 360$^{\circ }$ videos of forest or waterfall environment was able to decrease negative emotions such as fatigue and depression. In contrast, levels of fatigue were increased and self-esteem was decreased after exposure to urban environments. Also, in a study by Schutte et al.^57^ participants were exposed to a natural and an urban environment using 360$^{\circ }$ videos. Thereafter, participants reported significantly more restorativeness by exposure to the natural environment compared to the urban environment.

Multiple studies have reported stress recovery elicited by multisensory exposure to nature in VR. For instance, in a study by Annerstedt et al.^58^, participants experienced a psycho-social stress (i.e., TSST^59^) in VR followed by an exposure to natural scenes in VR either with or without sound. As a result, recovery from stress was facilitated by exposure to VR nature and was enhanced when the environment was presented with natural sounds. In another study by Hedblom et al.^60^ visual stimuli (i.e., 360$^{\circ }$ photos of urban, park, and forest environments) were accompanied by auditory stimuli (e.g., bird songs for natural environments) and olfactory stimuli (e.g., grass odour for park). Consequently, exposure to natural environments reduced stress levels significantly. Finally, Schebella et al.^61^ suggested that multisensory exposure to 360$^{\circ }$ videos of nature are beneficial to recovery from stress compared to visual-only exposure and that recovery is least effective in a virtual urban environment.

In order to use visual representation of natural environments in experiments or for preventive and/or therapeutic purposes, it is important to know whether the level of immersion and its associated feeling of presence are decisive for the extent of the effect. Different levels of immersion could be, for example: the actual stay in a natural environment, viewing a natural environment through a window, viewing a 360$^{\circ }$ video of a natural environment (stereoscopic or monoscopic) on a display (such as a smartphone with integrated gyroscope or using a head-mounted display (HMD)), staying in an artificially generated virtual world or watching a regular video or pictures of nature. To subjectively distinguish between different levels of immersion in VR context, the sense of presence is usually measured. It describes the psychological sense of being in a virtual environment^42^ and can have multiple components such as the sense of being physically present in a place (spatial presence), the attention devoted to the virtual environment (experienced involvement), as well as the experienced realism of the environment^62^.

Previous studies have examined different levels of immersion based on human’s psycho- and physiological responses^63–72^. For instance, a study^63^ suggested that closest to reality psychological responses can be achieved by a 360$^{\circ }$ panorama and physiological responses by a 3D model of the real environment (an interior shopping environment). Although in the same study, different levels of immersion including a conventional photograph of the environment were employed, they were all compared against the real environment and not against one another. In another study^65^ a significant increase in the sense of presence from monoscopic to stereoscopic and from 180$^{\circ }$ to 360$^{\circ }$ images was demonstrated. In addition our group, Forlim et al.^68^ previously reported that stereoscopic renderings delivered via an HMD elicit higher functional connectivity in the brain when compared to monoscopic renderings on projection screens or HMDs. Furthermore, Chirico et al.^72^ confirmed that immersive videos enhance the intensity of self-reported awe emotion as well as parasympathetic activation compared to 2D screen videos.

However, to the best of our knowledge, a direct comparison between conventional photo presentations and 360$^{\circ }$ video presentation of nature has not been tested so far. Hence, it is largely unknown whether conventional photo presentations suffice to create the full impact of virtual exposure to nature or whether an immersive display such as 360$^{\circ }$ video presentation can further increase the positive effects.

**Figure 1:**
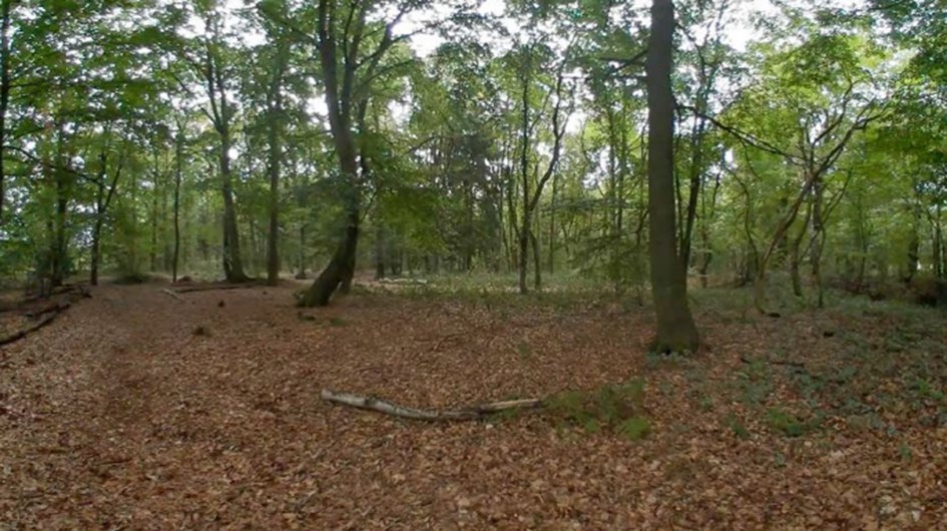
Forest environment.

**Figure 2:**
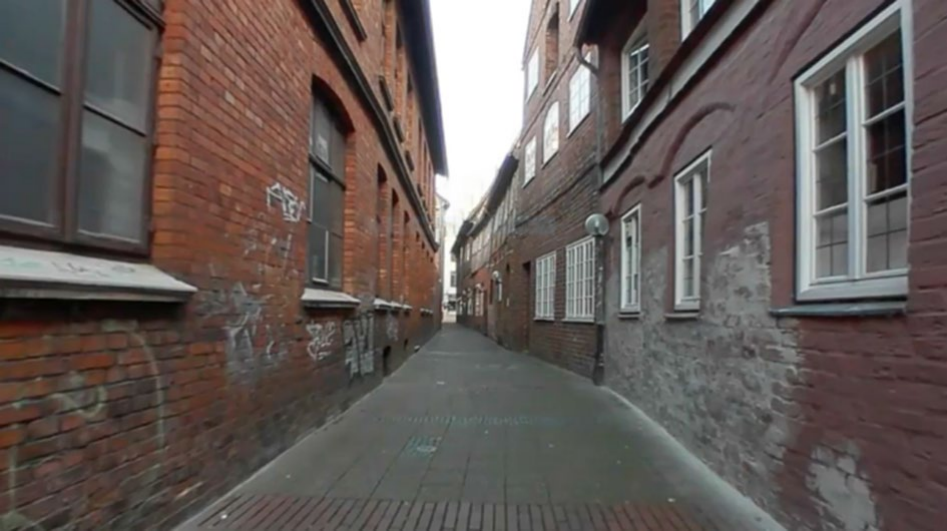
Urban environment.

**Figure 3:**
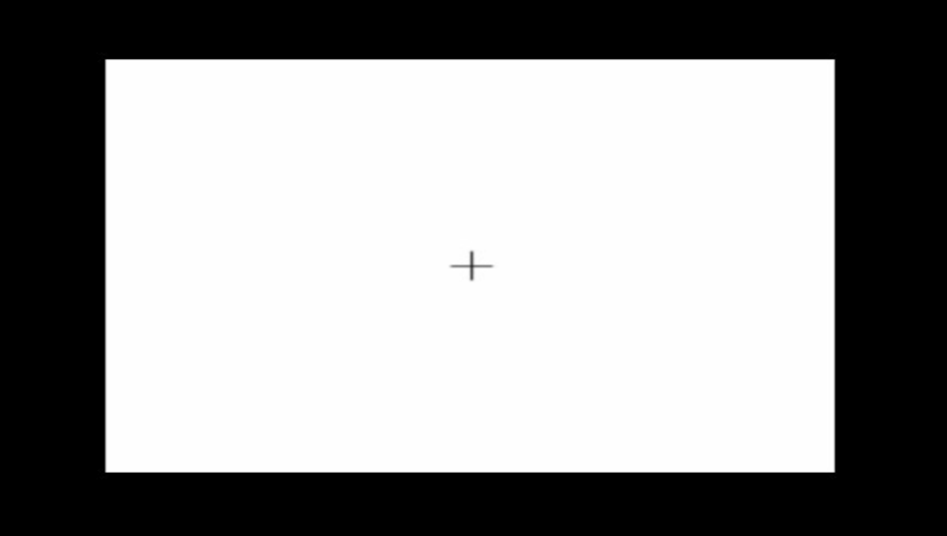
Control environment.

**Figure 4:**
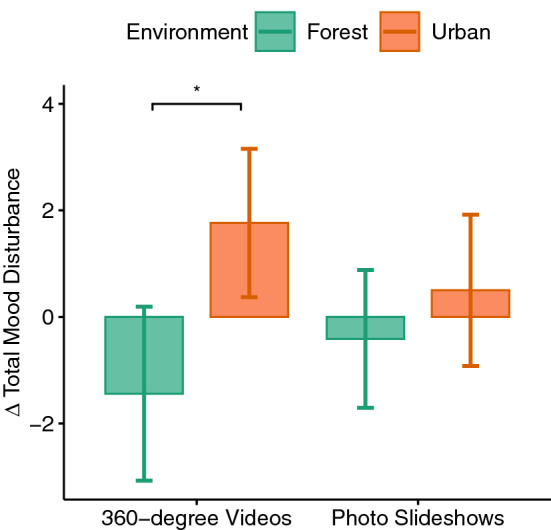
Difference scores of the total mood disturbance.

**Figure 5:**
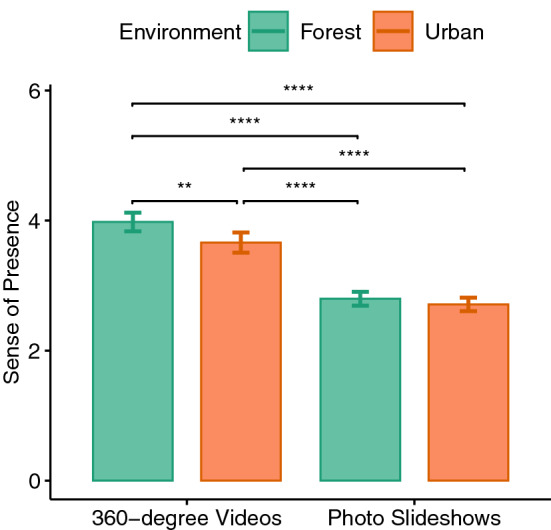
Sense of presence.

The experiment followed a within-subject design and consisted of a control (in a silent black virtual room with a white screen in the middle showing a fixation cross) and four experimental conditions (see Fig. 12). Each experimental condition consisted of three parts: (2) a cognitive test (serially subtracting 13 from a given starting number such as 1022) for 5 min, (2) exposure for 6 min to either an urban (i.e., an old town of northern Germany, see Fig. 2) or a forest (i.e., a northern German mixed forest, see Fig. 1) virtual environment presented either using 360$^{\circ }$ videos or conventional photo slideshows from the same content both displayed via an HMD, and lastly (3) filling out the questionnaires. The order of the experimental conditions was counterbalanced. The control condition was consistently administered at the beginning and immediately after the baseline measurement (see “Methods”***). During the experiment physiological data was recorded, namely galvanic skin response (GSR) and HR. It is worth noting that the cognitive test in this experiment served two functions simultaneously: it was used to induce stress prior to exposure and at the same time to measure cognitive performance due to the prior exposure phase. That is, the cognitive test measured the cognitive performance after exposure to the previous (and not following) condition. Thus, after the last condition, the cognitive test was administered for the last time measuring the cognitive performance after the last exposure.

## Results

To analyse the responses of our mood and stress-related questionnaires (i.e., STADI-S, POMS, SSSQ, and PSS) as well as the cognitive test, differences between experimental conditions and the control measurements were computed. On these difference scores, a two (environment: forest, urban) by two (immersion: 360$^{\circ }$ videos, conventional photo slideshows) repeated-measures ANOVA was performed.

### Questionnaire data

#### State-Trait Anxiety Depression Inventory-State (STADI-S)

No significant effect of the factors was found neither for depression nor anxiety sub-scales of the state version of the STADI (STADI-S).

#### Profile of mood states (POMS)

The environment factor (i.e., the type of environment: forest vs. urban) showed a significant negative effect on mood ($F(1,33)=5.02, p=0.03, \eta _p^2=0.13$). Paired t tests suggested that exposure to the urban environment disturbed the participants’ mood more than the forest environment ($p=0.027$) and that this difference was significant between the 360$^{\circ }$ videos of forest and urban environments ($p=0.028$, see Fig. 4). A main effect of the immersion level or its interaction with the environment factor could not be observed. We also calculated the difference scores (exposure-control) for each sub-scale of POMS and performed the two-way ANOVA. A significant main effect of environment could be found for fatigue only ($F(1,33)=5.19, p=0.03, \eta _p^2=0.13$). While the 360$^{\circ }$ videos of forest decreased the feeling of fatigue, pairwise comparisons revealed that this reduction was significantly different from the changes in fatigue (i.e., increase of fatigue) elicited by the 360$^{\circ }$ videos ($p=0.027$) or photo slideshows of the urban environment ($p=0.016$). No significant difference was observed between photo slideshows and 360$^{\circ }$ videos of the forest environment.

#### Short Stress State Questionnaire (SSSQ)

No significant main or interaction effects of the environment and immersion factors were found for neither of the SSSQ sub-scales namely task engagement, distress, nor worry.

#### Perceived Stress Scale (PSS)

No significant main or interaction effects of the environment and immersion factors could be found for the PSS score.

#### Igroup Presence Questionnaire (IPQ)

The IPQ scores of each condition were directly used for the analysis, without the control measurements being subtracted from them. As a result, a significant main effect of immersion level could be observed for the sense of presence ($F(1,33)=79.11, p<0.001, \eta _p^2=0.706$). The sense of presence for the 360$^{\circ }$ videos was higher than the slideshow conditions ($p<0.001$). The environment factor also had a significant effect on the sense of presence ($F(1,33)=13.927, p<0.001, \eta _p^2=0.297$) in such a way that the forest environment induced a higher sense of presence compared to the urban environment ($p<0.001$). No significant interaction effect was found (see Fig. 5).

For the sense of being there (or the general presence) sub-scale, we found a significant main effect of immersion level ($F(1,33)=62.767, p<0.001, \eta _p^2=0.655$), which shows that the mean value of the 360$^{\circ }$ videos was higher than the mean value of the slideshows ($p<0.001$).

For the spatial presence, the main effect of immersion level was significant ($F(1,33)=96.371, p<0.001, \eta _p^2=0.745$). Here, the mean value of the 360$^{\circ }$ videos was higher than the mean value of the slideshows ($p<0.001$). Moreover, our results show a significant main effect of the factor environment ($F(1,33)=11.85, p=0.002, \eta _p^2=0.264$) and therefore underline that the mean value of the forest environment was higher than the urban environment ($p<0.01$). Therefore, the 360$^{\circ }$ video and the forest environment led to a higher sense of spatial presence.

For the involvement sub-scale, the main effects of immersion level ($F(1,33)=19.649, p<0.001, \eta _p^2=0.373$) as well as the environment ($F(1,33)=10.574, p=0.003, \eta _p^2=0.243$) were significant. The 360$^{\circ }$ videos ($p<0.001$) and the forest environment ($p<0.01$) showed higher involvement values compared to respectively slideshows and the urban environment. In addition, a significant interaction effect of these two factors could be observed ($F(1,33)=35.254, p<0.001, \eta _p^2=0.517$). Here the highest involvement values were observed in the 360$^{\circ }$ forest video ($p<0.001$).

For the experienced realism, the main effect of the immersion level was significant ($F(1,33)=17.006, p<0.001, \eta _p^2=0.34$) and the 360$^{\circ }$ videos had higher values than the slideshows ($p<0.001$).

#### Simulator Sickness Questionnaire (SSQ)

The SSQ was administered two times: once at the beginning of the experiment (i.e., prior to wearing the HMD for the first time) and once in the end (i.e., after the last experimental condition). A paired t test suggested a significant ($t(33)=3.67, p<0.001, d=.63$) increase of the total simulator sickness score from pre- ($M=14.08, SD=16.37$) to post measurements ($M=30.8, SD=28.33$). This means that the experiment and its total associated stay in VR increased the symptoms of simulator sickness.

### Physiological measures

Considering the cognitive test as a stress induction task prior to exposure, it can be seen in Fig. 10 that physiological arousal measured by the GSR values were increased during the cognitive test phase prior to the exposure and were decreased during the exposure for all four conditions. A three (experiment phase: baseline, (cognitive) test, exposure) $\times$ two (environment: forest, urban) $\times$ two (immersion level: 360$^{\circ }$ videos, photo slideshows) repeated-measures ANOVA showed a significant main effect of experiment phase ($F(2,66)=17.76, p<0.001, \eta _p^2=0.35$) and a significant interaction of immersion and experiment phase ($F(2,66)=3.38, p<0.05, \eta _p^2=0.09$). The results of pairwise t tests showed significant differences ($p<0.001$) between all three phases. Thus, the cognitive test could successfully serve its first function to induce stress prior to exposure.

Figure 11 depicts the mean HR values during different phases of the experiment for all four conditions. A three-way (experiment phase, environment, immersion level) repeated-measures ANOVA showed a significant main effect of experiment phase ($F(2,66)=13.51, p<0.001, \eta _p^2=0.29$). Pairwise comparisons suggest that participants experienced the lowest HR values during the exposure compared to the baseline ($p<0.001$) and cognitive test phase ($p<0.001$). The difference between the baseline and the cognitive test phase was not significant probably due to ceiling effects. During the exposure, however, their HR decreased significantly.

**Figure 6:**
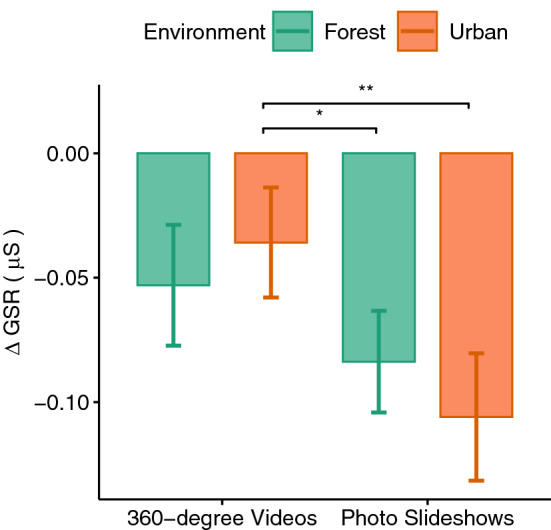
GSR difference scores.

**Figure 7:**
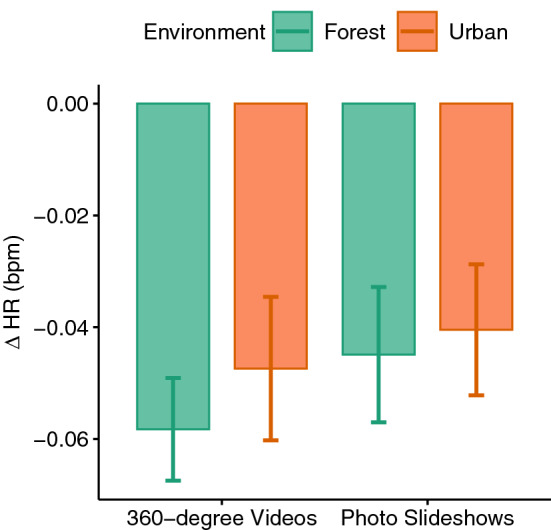
HR difference scores.

Difference scores were calculated for both physiological measures by subtracting the mean values during the cognitive test phase from the mean values during the exposure phase (see Figs. 6, 7). A two-way repeated measures ANOVA for the factors environment and level of immersion showed a significant effect of the immersion level for the GSR values ($F(1,33)=8.55, p<0.01, \eta _p^2=0.21$). A paired t test suggested that the GSR difference scores were significantly larger ($p<0.01$) for the photo slideshow conditions compared to the 360$^{\circ }$ video conditions. Pairwise comparisons showed that urban ($p<0.01$) and forest ($p<0.05$) photo slideshows caused larger difference scores compared to 360$^{\circ }$ videos of the urban environment (see Fig. 6). The pairwise comparisons did not show any significant difference between the 360$^{\circ }$ videos of the forest environment and any other conditions. No significant main or interaction effect could be observed for the HR difference scores. That is, all four conditions decreased HR with no significant difference.

**Figure 8:**
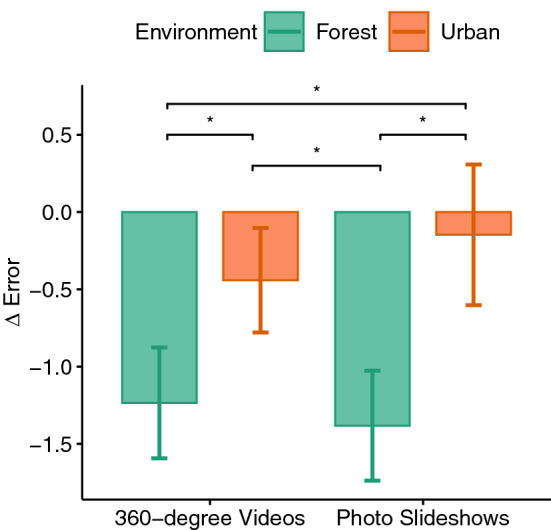
Difference scores of the cognitive test errors.

**Figure 9:**
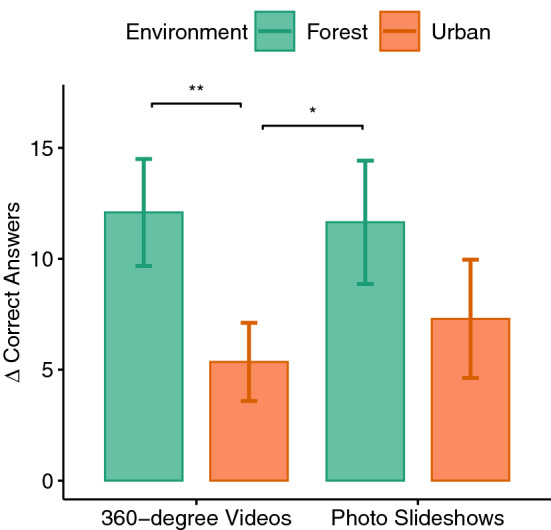
Difference scores of the cognitive test consecutive correct answers.

**Figure 10:**
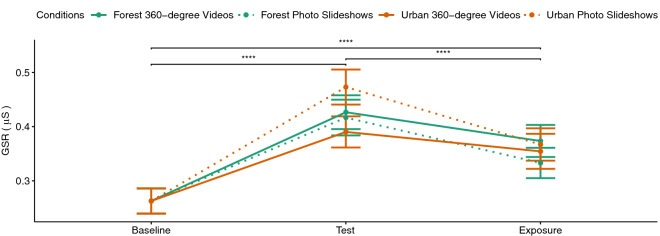
GSR measures during different phases of the experiment. The four conditions are distinguished by the line color (green for forest and orange for urban environment) and the line type (dotted for slideshows and solid lines for 360$^{\circ }$ videos).

**Figure 11:**
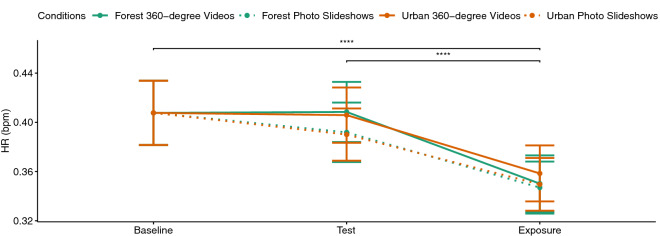
HR measures during different phases of the experiment. The four conditions are distinguished by the line color (green for forest and orange for urban environment) and the line type (dotted for slideshows and solid lines for 360$^{\circ }$ videos).

**Figure 12:**
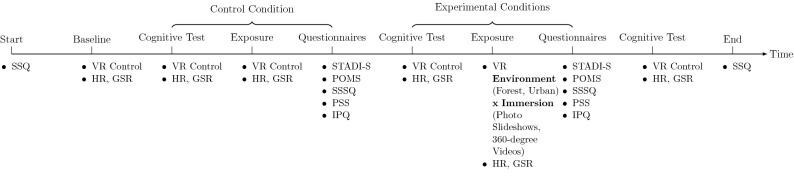
Experimental procedure. *VR* virtual reality (i.e., the contents were displayed using a head-mounted display), *Control* a silent black virtual room with a white screen in the middle showing a fixation cross, *SSQ* Simulator Sickness Questionnaire, *HR* Heart Rate, *GSR* Galvanic Skin Response or Skin Conductance Response (SCR), *STADI-S* State Trait Anxiety Depression Inventory-State, *POMS* Profiles of Mood States, *SSSQ* Short Stress State Questionnaire, *PSS* Perceived Stress Scale, *IPQ* Igroup Presence Questionnaire.

### Cognitive test

The cognitive test was considered as a dependent variable measuring the cognitive performance after the exposure phase. The environment factor had a significant effect on the errors ($F(1,33)=9.52, p=0.004, \eta _p^2=0.22$) and the consecutive, correct answers given ($F(1,33)=13.56, p<0.001, \eta _p^2=0.29$) in the cognitive test acquired after exposure. Paired t tests revealed that the number of errors (see Fig. 8) in the forest environment was significantly lower than in the urban environment ($p<0.001$) and the correct, consecutively given answers (see Fig. 9) were higher in the forest environment compared to the urban environment ($p<0.001$). No significant main effect of immersion or its interaction with the environment factor could be observed.

## Discussion

We hypothesized that the environment and the immersion level, as well as their interaction, have an influence on mood, stress recovery, and cognitive performance. In particular, we expected that the forest environment would produce a more positive effect than the urban environment. In addition, we hypothesized that more immersive presentations (i.e., 360$^{\circ }$ videos) create a higher sense of presence and consequently have greater effects, as more realistic environments would lead to realistic behavior and trigger corresponding responses^42^.

The effects of exposure to forest or urban environments on mood was measured by means of total mood disturbance. Here, it could be shown that the type of environment was determinant for the mood disturbance as exposure to the urban environment led to a significant mood disturbance whereas exposure to the forest environment resulted in a reduction of mood disturbance. In particular, the feeling of fatigue was increased after exposure to the urban environment regardless of their type of presentation (i.e., 360$^{\circ }$ videos or photo slideshows) and was reduced by exposure to the 360$^{\circ }$ videos of forest. This result confirms the findings of previous studies^47,54,56,57,61,73^. Despite variations in visual and auditory stimuli of the previously studied urban environments in VR, they all reported mood disturbances; whether the urban environment was a crowded subway station^56^, or shopping mall^61^ or shopping plaza^56^, or a small town with buildings lining streets, some road traffic, a pedestrian mall, and the sound of traffic and people talking in the pedestrian mall^57^. We intentionally excluded crowds, cars, and prominent nature elements such as trees. Thus, our VR urban environment comprised of only buildings as visual stimuli and mostly the sound of wind breeze as auditory stimuli. Yet exposure to this environment disturbed our participants’ mood. On the other hand, our forest environment was successful in reducing the disturbed mood. Since no significant effect of immersion level on mood could be shown in this experiment, it can be stated that photos of the environment are sufficient to observe its effects on mood. Nevertheless, the feeling of fatigue could be decreased only by exposure to 360$^{\circ }$ videos of the forest. Therefore, although Browning et al.^47^ could show an improved mood for the real exposure to outdoor forest only, our findings proved that VR exposure to forest can be beneficial for inducing positive mood. This is in line with the findings of previous studies that showed exposure to VR nature can improve mood and reduce negative affect, whether the VR nature was 360$^{\circ }$ videos of rural areas and remote beaches^54^ or various types of forest environments^53,56^.

No significant effects were found for STADI-S, PSS, and SSSQ. Since we already showed that the urban environment caused mood disturbance, one could expect that the STADI-S shows a similar effect. However, it should be noted that STADI-S rather covers more clinical aspects such as anxiety and depression, whereas the POMS measures mood changes in a more healthy range. Therefore, a possible explanation would be that the healthy volunteers did indeed experience a disturbance in mood by being exposed to the urban environment. However this mood disturbance was not strong enough to be detected by the scales of the STADI-S.

An increase of the physiological arousal measured by the GSR values could be observed from the baseline to the cognitive test phase which was again decreased by exposure to any of our four experimental conditions. The photo slideshows in this case were more effective in lowering the arousal levels compared to 360$^{\circ }$ videos. The reason here could be the higher immersion level of 360$^{\circ }$ videos and their associated sense of presence which has been shown to be positively correlated with physiological arousal^74,75^. The immersive VR has been also shown effective in inducing emotions (such as the feeling of awe) and enhancing their intensity^72^. Thus compared to non-immersive photos, immersive videos may elicit higher emotional reactions which again results in higher physiological responses. Therefore, although 360$^{\circ }$ videos were able to reduce the physiological arousal, their higher immersion level prevented this arousal reduction to reach the same level as the non-immersive photo slideshows. Thus, to reduce physiological arousal caused by psycho-social stressors, one should rather use conventional photos of either urban or forest environments.

Besides GSR, we measured participants’ HR during the course of experiment. The results showed that the HR was already high at baseline and the cognitive test did not increase it any further. A limitation of this study is that we did not plan any additional resting phase before the baseline measurement started. Perhaps this is the reason why the difference between the HR measurements at the baseline and during the test phase was not significant, probably due to ceiling effects. The exposure to any of our conditions, however, was able to reduce the HR significantly, regardless of the type of environment or the level of immersion. This finding is in line with previous studies^54,56^. For instance, Yu et al.^56^ showed that blood pressure and HR were reduced by exposure to both urban and natural environments with no significant differences between them. Also, Anderson et al.^54^ showed that HR variability was reduced during the exposure to natural and indoor VR environments with no clear differences across them. In their study, the GSR values were also decreased for all conditions but this reduction was greater for the natural scenes, similar to the findings of Hedblom et al.^60^. Moreover, Schebella et al.^61^ showed that recovery from stress measured by HR values could be achieved by VR exposure to both natural and urban environments with no significant difference between them. Nevertheless, it is important to determine whether and which stimuli are best suited to decrease the induced stress. By analyzing the GSR values, we could show that all our stimuli could reduce the induced stress, but non-immersive stimuli were more effective in doing so.

The hypothesis that exposure to forest environment improves cognition was confirmed by this study. It could be shown that the maximum number of correct answers (number series) in two conditions exposing to the forest environment was higher and the total number of errors was lower. This can be attributed to the positive effect of exposure to the forest environment and cognitive benefits of interacting with nature which has been studied before^4,5,8,9,13,55,76,77^. For instance, Berman et al.^76^ found that viewing pictures of natural scenes can improve cognitive performance compared to urban scenes. Also, Chung et al.^55^ showed that 360$^{\circ }$ videos of nature can restore involuntary attention. In our study however, neither an effect of immersion level nor an interaction with the environment factor could be found. Therefore, it can be concluded that the presentation of forest using both methods namely the photo slideshows and the 360$^{\circ }$ videos had a positive effect on cognition with no significant difference between them. Thus, to induce a positive effect on cognition, a presentation of forest using the conventional photo slideshows might be enough to produce the full impact of forest exposure.

The cognitive test in this experiment served two functions: as a stress induction task prior to exposure and at the same time as a dependent variable measuring the cognitive performance after exposure. Initially, we had planned to use an additional cognitive test to measure the cognitive performance. However, during the piloting phase, we realized that the total length of the study could overwhelm the participants. Therefore, we used only one cognitive test to keep the length of the experiment limited to a reasonable time. This is a limitation of this study and in future work, these two functions could be disentangled from each other.

The results of the SSQ suggested that the experiment increased the symptoms of simulator sickness. A reason could be that the study was 180 min long during which a cognitive task was carried out repeatedly. Therefore, participation in the study could have led to fatigue and may have caused or exacerbated the symptoms of simulator sickness. Therefore, the symptoms can and should not be attributed solely to the exposure to the virtual environments. Moreover, a limitation of this study is that the SSQ was not administered after each condition. Thus, it cannot be determined whether different levels of immersion or types of environment played a role in inducing simulator sickness. Whether potentially associated simulator sickness prevented the positive effects of nature to occur remains a topic for future research.

The hypothesis that the immersive 360$^{\circ }$ videos can facilitate the positive effects of nature onto mood, recovery after stress and cognition could not be demonstrated in this experimental setup. Nevertheless, the IPQ results showed that the 360$^{\circ }$ videos did induce a higher sense of presence compared to the slideshows. Also, the IPQ sub-scale involvement was highest in the 360$^{\circ }$ video of a forest. Therefore, the question arises whether the provided level of immersion for the 360$^{\circ }$ videos was sufficient for changing our affective and cognitive measures. The 360$^{\circ }$ videos of this study were taken in a resolution of 4K and were monoscopic (i.e., the same image was displayed on both lenses). Monoscopic images lack cues of depth perception that affect the sense of spatial perception^78^. Since realistic representations have an impact on immersion^42^, the use of a 360$^{\circ }$ camera with a higher resolution (to render more realistic stimuli such as the movement of single leaves’) and stereoscopic display (i.e., different images shown on the respective lenses to create a sense of spatial depth) should be considered in future investigations. It might still be true that with stronger immersion and sense of presence, the reactions of the participants could have been more different between 360$^{\circ }$ videos and the photo slideshow.

However, in the present setup an advantage of using 360$^{\circ }$ videos compared to photos could not be determined, the positive influence of natural environments on cognition and reduction of mood disturbance could be observed. As the use of visual representations of natural environments can be a viable option in contexts that offer little access to natural resources. In future work, the underlying elements of the forest environments that cause the more positive impact in contrast to the urban environments should be further studied. It would be possible that it is not the forest in its complexity that is necessary to trigger the observed positive effects, but rather some bottom-up visual features that are commonly found in nature pictures. Previous research has shown that preference ratings of nature pictures can be explained by such lower-level image features^79,80^.

The Prospect-Refuge Theory^81^ suggests that humans have preferences for certain environments. According to Appleton^81^, humans prefer places that offer a safe and sheltered refuge and at the same time a good view or overview of the surrounding environment. This theory relies on evolutionary approaches, which require a predator to be able to observe a potential prey without being discovered. Accordingly, there is the possibility that there may be a natural preference for the environment of a dense forest over an empty road or a rather open space within a city. Consequently, future studies should take these aspects into account while selecting the virtual environments to be compared.

In this work, the visual stimuli of natural and urban environment were accompanied by the respective auditory stimuli recorded from that environment. In other words, while seeing either 360$^{\circ }$ videos or photo slideshows of forest environment, our participants could listen to the sound of birds singing in that forest. The selected urban environment for this study was an empty old town in which mostly a soft wind breeze could be heard. As mentioned earlier, our main reason for including the auditory stimuli was to increase the feeling of presence^42,82^. However, since Annerstedt et al.^58^ showed that recovery from stress by exposure to VR nature was enhanced when the environment had nature sounds, one may consider to repeat the present experiment to investigate the effects of pure visual stimuli. Furthermore, in the 360$^{\circ }$ videos of the forest, subtle movements of the leaves of the trees caused by the wind breeze as well as the changes of the sunlight when shined through the trees were observable. Such subtle changes could indeed not be observed in the urban environment. We consider this as a limitation of this work. Thus, future studies may decide to provide an urban scene with a comparable level of movement as the forest.

In addition, in this experiment, the conventional photo slideshows were displayed using a VR HMD which was required for presenting the 360$^{\circ }$ videos but not for the photo slideshow. On the one hand, using the HMD for both conditions enabled us to control for unintentional effects of the display medium while comparing the immersion properties of the presented materials (i.e., 360$^{\circ }$ videos or conventional photos), but on the other hand limited us from generalizing the findings to other display media. As presentation of conventional photo slideshows on a monitor may not produce the same effects as presenting them using an HMD and remains a topic for further research.

In sum, the benefits of interacting with real or virtual nature has been reported in previous studies. Virtual exposure to nature has been administered classically using conventional photos and recently using immersive 360$^{\circ }$ videos or computer generated models of nature. In this work, we aimed to answer the question of whether immersive 360$^{\circ }$ videos of nature intensify its positive effects on mood, stress recovery, and cognition compared to conventional photos of nature. Our results suggest that indeed exposure to photos of a forest environment suffice to prevent mood disturbance observed in response to urban exposure, reduce physiological arousal, and improve cognition. In addition, photos of either urban or forest environment were both more effective in reducing physiological arousal compared to immersive 360$^{\circ }$ videos. Thus, in contrast to our priori hypothesis, more immersive presentation of the forest environment could not lead to more positive effects of nature.

## Methods

### Participants

Recruitment of participants took place via an email distributor among the students of the Faculty of Computer Science at the University of Hamburg. In addition, the study was advertised on the campus of the University of Hamburg and the University of Applied Sciences in Hamburg and a call in social networks. A total of 35 subjects participated in the study. However, one person had to be excluded due to deuteranopia (green blindness). The remaining 34 subjects (11 female) were between 21 and 34 years old ($M=27.26, SD=4.144$). The study was approved by the local psychological ethics committee of the Center for Psycho-social Medicine at the University Hospital Hamburg-Eppendorf and was carried out in accordance with relevant guidelines and regulations.

### Materials

We selected a northern German mixed forest as the forest environment (see Fig. 1). Since our focus was on vegetation, other natural elements such as water and animals or humans were avoided and were not present in this environment. Our urban environment was an old town of northern Germany (see Fig. 2) which contained no vegetation nor animals or humans. Each 360$^{\circ }$ video was a 6 min video consisting of three 2 min single stationary videos. To shoot the individual videos, the tripod with the camera was moved 6 m forward, measured from the center of the tripod. The result is a composition of three stationary single shots in which the tripod, per single shot, was placed firmly in one place. Looking at the final video created the impression of teleportation between these three shots.

In total, three different environments were created for the study. The first environment was a black room with a white screen in its center. In the middle of the white screen was a black fixation cross (Fig. 3). This environment had no background sound and was used as the control condition. The second environment was identical to the first one, with the difference that the slideshows were played on the screen. The virtual camera in these two environments was one meter away from the virtual screen. The 360$^{\circ }$ videos were played in the third environment on the inner side of a virtual sphere. Here, the virtual camera was placed in the center of the sphere to create the impression of being inside the 360$^{\circ }$ VR environments.

The experiment was conducted in a laboratory room. The participants were seated on a firm chair. The position of the chair was fixed to ensure a fixed position in the virtual environment. During the experiment, the subjects wore a HTC Vive Pro HMD as well as Neulog Pulse and Galvanic Skin Response (GSR) sensors. GSR or skin conductance response (SCR) measures the amount of changes in electrical conductivity of the skin (in this study, at the finger of the non-dominant hand) when its glands produce ionic sweat in response to a given stimulus. Thus, it is considered as an indicator of localized phasic arousal processes and has been interpreted as an indicator of stress in the literature^83^. Additionally, previous research in particular on TSST^59^, suggest that both heart rate and heart rate variability can detect the physiological effects of stress on human participants. Therefore, we concluded that both measures apply as our cognitive test was taken from the TSST. In this study, we measured heart rate since it could successfully indicate physiological changes of performing multiple TSST in VR in a previous study^75^. For rendering, system control, and logging an Intel Computer (Core i7 6900 K at 3.2 GHz) with an NVIDIA GeForce GTX 1080 graphics card was used. The questionnaires were completed on a MacBook Pro (Retina, 13 inches, model year: end of 2013).

The following questionnaires were employed in this study:

**State Trait Anxiety Depression Inventory-State (STADI-S)**^84^ measures the current state of anxiety and depression of a person. It consists of 20 statements scoring from 1 (Not at all) to 4 (Very much so). The sub-scales of excitement and concern estimate the level of anxiety whereas the euthymia (positive mood) and dysthymia (negative mood) sub-scales are used as dimensions of depression.

**Profiles of Mood States (POMS)**^85^ was used to assess mood. For this purpose, a value for the total mood disturbance was determined. The questionnaire contains keywords and statements that describe different feelings and are scored on a 5-point Likert scale ranging from 1 (Not at all) to 5 (Extremely).

**Short Stress State Questionnaire (SSSQ)**^86,87^ records the status of engagement, distress and worry after a given task. The questionnaire consists of 24 items rated from 1 (Not at all) to 5 (Very much so).

**Perceived Stress Scale (PSS)**^88^ measures the perception of stress. It contains 10 items ranging from 0 (Never) to 4 (Very often). As the original items refer to the situations during the past month in one’s life, for the purpose of this study, the items were modified to measure the momentary perceived stress.

**Igroup Presence Questionnaire (IPQ)**^62^ was used to measure the perceived sense of presence in VR. It contains 14 items on a 7-point Likert scale ranging from 0 to 6 with different scale anchor, meaning that some items have general scale anchors (0: Fully disagree to 6: Fully agree) and some have more precise anchors (e.g., 0: Not consistent and 6: Very consistent). The questionnaire has also four sub-scales: general presence or the sense of being there, spatial presence, involvement and experienced realism.

**Simulator Sickness Questionnaire (SSQ)**^89^ measures 16 symptoms that may occur during or after VR exposure. The symptoms are rated from 0 (None) to 3 (Hard) and are classified into three categories: Nausea, Oculomotor and Disorientation.

#### Procedure

Upon arrival, the subjects were informed about the purpose of the study and their right to interrupt or quit at any time. Thereafter, they signed the informed consent. At baseline (see Fig. 12), the SSQ-pre was administered and the baseline GSR and HR were measured. For this purpose, the subjects wore the HMD and saw the control environment (i.e., a black virtual room with a white screen showing a fixation cross) for 6 min. Participants were asked to look at the fixation cross on the white screen and not to move or to speak.

The study had a within-subject design and consisted of a control and four experimental conditions. The order of the experimental conditions was counterbalanced but the control condition was consistently administered at the beginning and immediately after the baseline measurement. Each condition consisted of three parts.

In the first part, the participants were asked to serially subtract 13 from a given starting number (e.g., 1022) while wearing the HMD and seeing the control environment. They were told to generate correct, sequential answers as fast as possible. They did not receive any feedback for giving the correct answers but immediately after giving an incorrect answer they were asked to start from the beginning. This task was taken from the Trier Social Stress Test (TSST)^59^, a widely used protocol for inducing moderate psycho-social stress in laboratory settings. TSST consists of a 5-min free speech followed by another 5 min performing a mental arithmetic task. We employed the TSST’s mental arithmetic task as the first part of our conditions. We measured the number of correct and incorrect answers, the total number of answers as well as HR and GSR values. This part lasted for 5 min. When treating the performance in this task as the dependent variable we compared the cognitive performances after the respective exposure to one of the four experimental conditions.

In the second part, the participants were exposed for 6 min to either an urban or a forest virtual environment presented either using 360$^{\circ }$ videos or conventional photos taken from the same video in the form of a slideshow. In the control condition, the presented virtual environment did not change during the exposure. In other words, after performing the mental arithmetic task inside the control environment during the first part, the subjects were exposed further to the control environment and did not have any specific task to do. They were, however, allowed to look around in the virtual environment and were instructed not to speak or move. In this part, GSR and HR were measured.

In the third part and after the exposure, the participants took off the HMD and the sensors and filled out the following questionnaires: STADI-S, POMS, SSSQ, PSS, IPQ.

It is important to clarify that the cognitive test in this experiment was designed to test the participants’ cognitive performance after exposure to the previous (and not following) virtual environment. For this reason, after the last condition, the cognitive test was administered again. Thereafter the participants filled out the SSQ-post and were compensated with course credits.

### Data analysis

Prior to the analysis the physiological signals were smoothed using a low-pass Butterworth filter with the cutoff frequency of 1 Hz. Thereafter, they were normalized using the following formula^90,91^:

1 $$\begin{aligned} {{\tilde{S} } = \frac{ s - min ({\bar{s} })}{max ({\bar{s} }) - min ({\bar{s} })} } \end{aligned}$$

where S is the raw signal, s the smoothed version of S, $\bar{s}$ the signal taken over the entire session, and $\tilde{S}$ the normalized signal.

Subsequently, difference scores were used, with the average values measured during the cognitive test being subtracted from the values of the exposure phase. On these difference scores the two-way repeated-measures ANOVA was performed.

In order to analyse the cognitive performance as well as the questionnaire data, the difference scores were calculated by subtracting the respective value from the control condition from the values of the given experimental subjective or cognitive measure. These differences were not calculated for the IPQ responses as we were interested in the original presence values after each condition and not the changes with respect to the control condition.

It has to be noted that according to the Shapiro–Wilk test, some data were normally distributed (e.g., IPQ Sense of Presence as well as GSR and HR Difference scores) and some were not (e.g., Cognitive Test). Therefore, we decided to report the analysis based on parametric tests in order to not switch between statistical tests. Thus, to test our hypotheses, for all data except the SSQ, two-way repeated-measures ANOVAs were performed, with a significance level at 0.05. As an effect strength, the partial eta squared ($\eta ^2$) was reported. Thereby, a value of 0.01 represents a small effect, 0.06 a medium effect, and 0.14 a large effect^92^. The SSQ responses were analysed using a paired t test with a significance level of 0.05. Additionally, Cohen's d was reported as the effect size for t-test which is commonly interpreted as small (d = .2), medium (d = .5), and large (d = .8) effects^92^.

## Data Availability

Upon request the authors will make the data available .
